# Participant Adherence in Repeated-Dose Clinical Studies Using Video-Based Observation: Retrospective Data Analysis

**DOI:** 10.2196/65668

**Published:** 2025-04-07

**Authors:** Jihong Song, Sungpil Han, Suein Choi, Jonghyuk Lim, Byeong Yeob Oh, Dongoh Shin, Seunghoon Han

**Affiliations:** 1 Department of Pharmacology College of Medicine The Catholic University of Korea Seoul Republic of Korea; 2 Department of Clinical Pharmacology and Therapeutics Seoul St. Mary’s Hospital, College of Medicine The Catholic University of Korea Seoul Republic of Korea; 3 CareSquare, Inc. Seoul Republic of Korea

**Keywords:** adherence, mobile health, self-administration, repeated-dose clinical trials, video-based monitoring, mobile phone

## Abstract

**Background:**

Maintaining accurate medication records in clinical trials is essential to ensure data validity. Traditional methods such as direct observation, self-reporting, and pill counts have shown limitations that make them inaccurate or impractical. Video-based monitoring systems, available as commercial or proprietary mobile applications for smartphones and tablets, offer a promising solution to these traditional limitations. In Korea, a system applicable to the clinical trial context has been developed and used.

**Objective:**

This study aimed to evaluate the usefulness of an asynchronous video-based self-administration of the investigational medicinal product (SAI) monitoring system (VSMS) in ensuring accurate dosing and validating participant adherence to planned dosing times in repeated-dose clinical trials.

**Methods:**

A retrospective analysis was conducted using data from 17,619 SAI events in repeated-dose clinical trials using the VSMS between February 2020 and March 2023. The SAI events were classified into four categories: (1) Verified on-time dosing, (2) Verified deviated dosing, (3) Unverified dosing, and (4) Missed dosing. Analysis methods included calculating the success rate for verified SAI events and analyzing trends in difference between planned and actual dosing times (PADEV) over the dosing period and by push notification type. The mean PADEV for each subsequent dosing period was compared with the initial period using either a paired *t* test or a Wilcoxon signed-rank test to assess any differences.

**Results:**

A comprehensive analysis of 17,619 scheduled SAI events across 14 cohorts demonstrated a high success rate of 97% (17,151/17,619), with only 3% (468/17,619) unsuccessful due to issues like unclear video recordings or technical difficulties. Of the successful events, 99% (16,975/17,151) were verified as on-time dosing, confirming that the dosing occurred within the designated SAI time window with appropriate recorded behavior. In addition, over 90% (367/407) of participants consistently reported dosing videos on all analyzed SAI days, with most days showing over 90% objective dosing data, underscoring the system’s effectiveness in supporting accurate SAI. There were cohort differences in the tendency to dose earlier or later, but no associated cohort characteristics were identified. The initial SAI behaviors were generally sustained during the whole period of participation, with only 16% (13/79) of study days showing significant shifts in actual dosing times. Earlier deviations in SAI times were observed when only dosing notifications were used, compared with using reminders together or no notifications.

**Conclusions:**

VSMS has proven to be an effective tool for obtaining dosing information with accuracy comparable to direct observation, even in remote settings. The use of various alarm features and appropriate intervention by the investigator or observer was identified as a way to minimize adherence deterioration. It is expected that the usage and usefulness of VSMS will be continuously improved through the accumulation of experience in various medical fields.

## Introduction

Repeated dose studies in healthy volunteers are essential for obtaining basic information about the pharmacokinetics of a drug. These studies are performed during the development of a specific investigational medicinal product (IMP) for which the dose of the active ingredient, formulation and route of administration has been determined. They include repeated dose escalation studies, drug-drug interaction studies, and some bioequivalence studies [1-3]. The changes in drug concentration that occur when an active ingredient is administered using a specific IMP are usually described in terms of the time elapsed after administration, from which various pharmacokinetics relationships are defined. Therefore, whether the IMP was actually administered and the time of administration are considered pivotal information to be obtained in such studies [4].

Historically, a variety of techniques have been used to ensure precise dosing information in clinical trials. These include direct observation by investigators, self-reporting through diaries or questionnaires, and pill counts [4,5]. Although direct observation is the most reliable method, it may be impractical and burdensome due to the necessity of frequent hospital visits, which increases costs and inconveniences participants [4,6-10]. Self-reporting methods are susceptible to biases such as recall and social desirability biases, which can lead to inaccuracies [5,6,11-15]. While pill counts are a straightforward approach, they cannot confirm actual administration, making them less reliable for studies requiring precise dosing records [12]. The inherent limitations of existing methodologies have consistently underscored the necessity for novel approaches capable of addressing the demands of repeated-dose studies.

The advent of mobile technologies has brought about innovative methodologies for the monitoring of the IMP administration in clinical trials [4,16]. One such advancement is the use of video-based observation, which permits the remote assessment of self-administration of the investigational medicinal product (SAI) [4]. Video-based observation uses either synchronous or asynchronous methodologies. In the former, the investigator observes the SAI in real-time [6,16-21], whereas in the latter, the participant uploads a video of the SAI for subsequent review [4,6,16,17,22-25]. This technology offers a degree of verification that is comparable to that of direct observation while reducing the necessity for frequent hospital visits, thus enhancing the convenience of participants and reducing the costs associated with the trial [4].

This study was to assess the usefulness of an asynchronous video-based SAI monitoring system (VSMS) through a simple and straightforward outcome measure: “Was the investigator able to reliably verify SAI using the videos uploaded by the participant?” This outcome measure is ultimately a direct indicator of whether the system performed as targeted, and was considered to be a composite of technical influences such as system reliability and sociodemographic influences such as digital literacy. In addition, if the results showed that the VSMS was sufficient to verify SAIs, it was planned to explore participants’ adherence to planned SAI times when using these systems. These evaluations will enhance our understanding of the validity and reliability of asynchronous VSMS in a range of repeated-dose trial settings.

## Methods

### The System Overview

The VSMS used in this study comprises a web-based system for investigators and a mobile app for participants. The web-based system allows investigators to (1) search for a QR code to match a participant to a specific participant number in the study; (2) view the SAI schedule, which should be assessed by calendar date; (3) view SAI-related videos uploaded by the participant; (4) determine if a valid administration is made and enter corresponding data; and (5) send the participant feedback on the SAI.

The system is designed to allow the investigator to make the final confirmation about the SAI results, with outcomes recorded in one of four categories (1)-(4):

(1) **Verified on-time dosing**

- The actual dosing time Provided by the system is within the allowed SAI time window and the dosing behavior recorded in the video is appropriate.
- The actual dosing time Provided by the system is outside the limits of the allowed SAI time window, but based on the video, it is verified that the appropriate dosing behavior occurred within the window (eg, video recording started before the time window range, but the actual dosing occurs within the window). In this case, the investigator manually corrected the actual dosing time.

(2) **Verified deviated dosing**

The actual dosing time provided by the system is outside the limits of the allowed SAI time window, and the appropriate SAI behavior is verified based on the video, but the timing is outside the window.

(3) **Unverified dosing**

SAI cannot be verified via video due to technical issues, participant nonadherence to recording procedures (eg, too dark recording environment, skipping oral cavity disclosure), and so on, but there is evidence to believe the participant performed the SAI (eg, the participant’s statement from a phone call with the reason of inappropriate video recording)

(4) **Missed dosing**

SAI cannot be verified via video and no evidence that the participants performed SAI can be obtained.

The mobile app is designed with a simple and user-friendly interface. The system may provide push notifications to participants before or after the scheduled dosing time, as specified in the protocol. Upon opening the application, the participant is immediately instructed to start the video recording and proceed with the SAI. The server connection time, recording start time, and recording end time were always captured based on the participants’ mobile device time to ensure that the data remained accurate even in situations such as slow internet speeds or transmission failures. In these cases, the recorded video was sent to the server as soon as mobile network conditions improved. Of the stored time data, the recording start time was displayed on the investigator’s web system as the default value for the actual dosing time. The recorded video was temporarily cached on the participants’ mobile device and deleted once the transfer to the server was confirmed. The behavior of the system according to the SAI schedule and procedures, storage and management of SAI data, and processing of other communication-related information was handled through servers operated by the service provider, which complied with all applicable quality and regulatory standards (encrypting network communications, ensuring physical security, and restricting video access only to authorized users).

### Clinical Trials and Cohorts

The data presented in this study were derived from repeated-dose clinical trials in healthy volunteers who used DoseEase. These trials were conducted between February 2020 and March 2023 and the IMPs were all self-administrable formulations (oral or ophthalmic). Trials that included groups with 2 or more different SAI schedules were also included in the analysis, and each group was treated as a separate Cohort. The eligibility criteria for participants across all trials were consistent and included the following: age between 19 years and 55 years, a minimum weight of 50 kg for men and 45 kg for women, with a BMI between 18.5 and 24.9, and confirmation of healthy condition in a comprehensive medical examination, which included an assessment of medical history, physical examination, and laboratory tests. Participants were excluded if they exhibited significant active disease at the screening stage or a history of previous disease that could affect the administration and absorption of the IMP.

Before enrollment began for the clinical trial, the service provider received a protocol from the research staff members to set up the system that specified the following;

- The number of participants and the principle of participant numbering.
- Cohort categorization according to the dosing schedule.
- SAI schedules by cohort and overall dosing schedule.
- Allowable time deviations per SAI event.
- Plan to deliver push notifications per SAI event.

The finalized system was piloted and validated by the investigators before the start of the trial.

At the first participant visit, the investigator accessed the VSMS web, retrieved and printed a QR code for each participant number, which was then scanned by the participant with that number using his or her mobile device (this was a key procedure to ensure that each participant was correctly identified without obtaining additional sensitive personal information). This registered the participants device in the system at the same time as the VSMS application was installed on the participants mobile device. This procedure was followed by training, which included 1-2 mock SAIs to familiarize the participant with the system. The mock SAI underscored the significance of securely positioning the mobile device and executing all procedures associated with the SAI (eg, the IMP preparation with a specified volume of water, the oral administration, and the disclosure of the oral cavity after dosing for oral administration) within the boundaries of the recording screen. Participants were also instructed to protect their privacy by wearing appropriate clothing and recording the video in a place where their home environment was not visible. At the visit immediately before the scheduled SAI, the participant received the IMP for the number of scheduled SAIs before the next visit, after which the IMP was managed by the participant at a location other than the hospital.

On days when SAI was scheduled, participants were either alerted via push notification on the mobile application or not, as specified in the protocol. The timing (before the planned dosing time [dosing notification], after the planned dosing time [dosing reminder], or both) and frequency of these alerts varied by cohort. As trained in advance, at their own available time (preferably within the allowable SAI time window specified in the protocol), participants performed the SAI while recording a video. If mobile network conditions were adequate, the video was sent to the server as soon as it was recorded, at which point it was made available to the investigator. If the video was not uploaded after the planned dosing time, investigators were able to contact participants by phone to remind them to perform SAI or to check for any technical issues.

**Figure 1:**
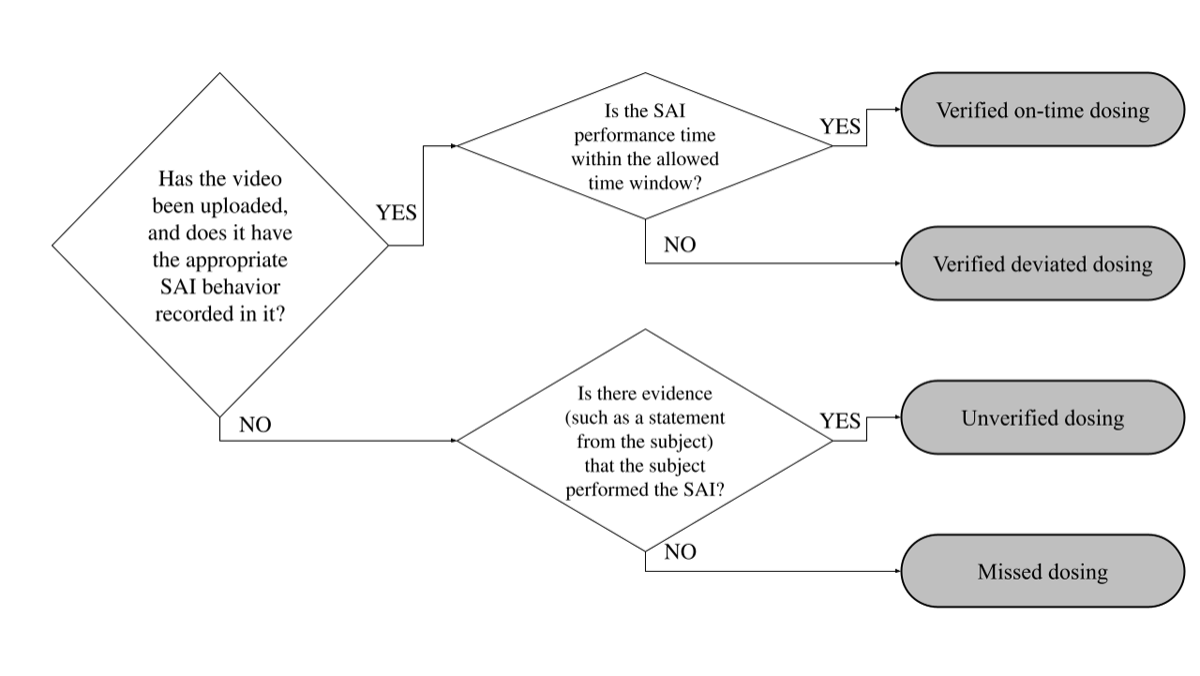
Investigator’s outcome decision-tree based on self-administered videos. SAI: self-administration of the investigational medicinal product.

For each SAI event, investigators evaluated the uploaded videos to assess whether the SAI was appropriate for each participant. A decision tree was provided to help the investigator confirm SAI videos and related records into the appropriate category (Figure 1). For cases other than verified on-time dosing, investigators were able to send proper feedback to the participant to improve adherence.

### Dataset for Statistical Analysis

Each SAI record included the planned Study Day (with the first dose day equal to 1), the Planned Dosing Time, the time the video recording started, and the investigator-confirmed actual dosing time. The difference between planned dosing time and actual dosing time (PADEV) was defined as the dosing time deviation. Study day values of 15 or more were recorded as “≥15” since the typical dosing duration was under 2 weeks. The investigator’s final decision on SAI (categorized as verified on-time dosing, verified deviated dosing, unverified dosing, or missed dosing) and the type of push notification delivery (categorized as none, dosing notification, dosing reminder, or both) were also included. All available data from participants who dropped out before completing all study procedures were included in the analysis. Dataset handling and statistical analyses were performed using R software (version 4.2.2; R Foundation for Statistical Computing). The significance level was set at .05, and all tests were 2-tailed.

### Fundamental Evaluation of Usefulness-Success Rate of the SAI Events

The primary goal of a VSMS is to ensure that the participant’s video-recorded SAI behavior is successfully transmitted to the investigator, who can then determine the SAI outcome. This goal cannot be achieved if the participant or investigator is unskilled in using the system, or if the data is incomplete due to technical problems with the system. Therefore, a VSMS may be considered “useful” when it adequately fulfills its intention of use. In this study, cases where the investigator’s final decision for the SAI is either verified on-time dosing or verified deviated dosing are collectively referred to as validated dosing. The percentage of validated dosing among all SAI records (referred to as the success rate, %) is evaluated as a measure of usefulness. The success rate was determined for each study day in which SAIs were performed in each cohort (considered separate cohorts if the same study included arms with 2 or more different dosing schedules). The success rate for each study day in the overall cohort and across all study days in each cohort were also calculated.

### Trends in Dosing Time Deviations by Cohort

VSMS allows investigators to view SAI records in real-time, unlike other existing methods. This means that investigator-participant interaction is possible in real time throughout the study period, with features such as push notifications (dosing notification or dosing reminder) and appropriate participant management to provide input on adherence. To assess the usefulness of this aspect, the trend of PADEV per study day was analyzed for each cohort. Only validated dosing data records from cohorts with the success rate of 90% or higher were used in this analysis (even verified on-time dosing records may have deviations between planned dosing time and actual dosing time). In the first analysis, a paired *t* test or a Wilcoxon signed-rank test (depending on the sample size) was performed on the difference between the mean of PADEV in the reference study days (the first 3 study days in which SAIs were performed) and the mean of PADEV in each subsequent study day to assess whether the dosing trend identified early in the dosing period was maintained thereafter. In the second analysis, we performed a descriptive statistical analysis of the trend in dosing deviations over the entire SAI period according to the type of push notification (type 1 [dosing notification only], type 2 [both dosing notification and dosing reminder], and type 3 [no push notifications]) provided, followed by *t* tests of means between groups.

### Ethical Considerations

This study used a retrospective SAI dataset gathered through the use of DoseEase (CareSquare, Inc), the inaugural asynchronous VSMS developed in Korea. Each individual clinical trial that provided data for this study was approved by the hospital’s institutional review board (IRB) before conduct and documented informed consent was obtained from all participants; however, this study is a secondary analysis of anonymized data already available. The dataset for this study included only SAI results interpreted by the investigators in each trial, and no personally identifiable information, including videos, was accessed or analyzed. We used the trial protocol number as the study identifier and the participant number assigned per protocol as the participant identifier. Due to the nature of this study, no additional consent was required for the individual participants who provided data in each trial, and the overall study design, including the consent waiver, was reviewed and approved by The Catholic University of Korea Songeui Campus IRB (MC23EASI0052, approval date: July 11, 2023).

## Results

### Cohort Characteristics

There were 10 trials included in the analysis based on the selection criteria, resulting in 14 unique cohort datasets. The Cohort characteristics could be summarized based on IMP characteristics, enrollment and completion rates, SAI schedule, push notification settings, and allowed time window for SAI. The route of administration was oral in 12 cohorts (86%, 12/14) and ophthalmic in 2 cohorts (14%, 2/14). The IMPs were formulated in various forms, including tablets, capsules, and powders. High completion rates were observed in 13 cohorts (93%, 13/14), with more than 80% of participants completing the study, and some cohorts achieving 100% completion (Table 1).

**Table 1:** Investigational medicinal product types and participant disposition of each Cohort.

Cohort	Dosing route	Formulation	Number of participants		
			Enrolled, n	Dropout, n	Completed n/N (%)
1	Oral	Tablet	90	8	82/90 (91)
2	Oral	Tablet	15	3	12/15 (80)
3	Oral	Tablet	23	4	19/23 (83)
4	Oral	Capsule or tablet	36	5	31/36 (86)
5	Oral	Tablet	40	9	31/40 (78)
6	Oral	powder	44	1	43/44 (98)
7	Oral	Capsule	8	1	7/8 (88)
8	Oral	Powder	45	0	45/45 (100)
9	Oral	Tablet	15	0	15/15 (100)
10	Oral	Tablet	15	2	13/15 (87)
11	Oral	Tablet	15	1	14/15 (93)
12	Ophthalmic	Eye drops	16	2	14/16 (88)
13	Ophthalmic	Eye drops	16	2	14/16 (88)
14	Oral	Tablet	29	2	27/29 (93)

The SAI schedules varied widely, ranging from 5 to 94 days, with daily frequencies varying from 1 to 4 times. In some cohorts, interinstitutional administration and SAIs were performed at different times on the same day. The planned dosing time was centered around a reference point (eg, 9 AM) for each cohort, with only minor variability in minutes across participants (eg, 9:02, 9:04, 9:06, etc). Push notifications were used in 11 cohorts (79%, 11/14), with the majority (73%, 8/11) using both dosing notifications and dosing reminders (Table 2).

**Table 2:** Self-administration of the investigational medicinal product-specific characteristics by cohort.

Cohort	SAI^a^ schedule					Dosing notification (min before the planned dosing time)		Dosing reminder (min after the planned dosing time)		Allowed time window for SAI (min)
	SAI day	Number of total SAI days	Number of SAIs per day	Planned dosing time^b^						
1	2-4, 6-8, 10-12, 14-16, 18, 19	14	1	9 AM	–60, –30, 0		15, 30, and 45		–60 to 60	
2	1, 2, 6, 7, 9-11	7	1	9 AM	N/A^c^		N/A		–60 to 60	
3	1, 2, 4-6, 10, 11	7	1	9 AM	N/A		N/A		–60 to 60	
4	1, 2, 12-15, 17-20, 24, 25	12	1	9 AM	N/A		N/A		–60 to 60	
5	1-4, 13-17, 21-23	12	1	9 AM	–60, –30, 0		15, 30, and 45		–60 to 60	
6	1-32	32	2	Variable^d^	–60, –15, 0		60 and 110		–120 to 120	
7	3, 5, 7, 9, 10, 12-14, 16-21, 23-30, 32-45, 47-60, 62-75, 77-90	78	1	9 AM	–30, –5, 0		10, 20, 165, and 665		–180 to 180	
8	1-28	28	2	Variable^d^	–60, –10, 0		60, 180, and 350		–120 to 360	
9	3-7	5	2^e^, 3	8 AM, 2 PM, and 8 PM	–30, 0		N/A		–120 to 120	
10	4-8, 11	6	2^e^, 3	8 AM, 2 PM, and 8 PM	–30, 0		N/A		–120 to 120	
11	5-9, 12	6	2^e^, 3	8 AM, 2 PM, and 8 PM	–30, 0		N/A		–120 to 120	
12	2-4, 8-96	92	1^e^, 2^e^, 4	1 AM, 9 AM, 9:10 AM, 5 PM	–10, 0		10		N/A	
13	1-89, 92-96	94	2^e^, 3	1 AM, 9 AM, 5 PM	–10, 0		10		N/A	
14	2, 3, 5, 6, 8, 9, 11	7	1	9 AM	–30, 0		30		–60 to 60	

^a^SAI: self-administration of the investigational medicinal product.

^b^The presented data refers to the reference point for the planned dosing time (eg, 9:00 AM) in each cohort, with minor variability across participants (eg, 9:02, 9:04, 9:06, etc).

^c^N/A: not applicable.

^d^The planned dosing times vary across participants, and no common reference point can be provided.

^e^SAI event and dosing by investigator performed on the same day.

### Fundamental Evaluation of the Usefulness-Success Rate of the SAI Events

A comprehensive analysis was conducted on 17,619 scheduled SAI events across the 14 cohorts to evaluate the system’s ability to fulfill its intended use. The analysis revealed a high success rate of 97% (17,151/17,619), while 3% (468/17,619) were unsuccessful due to issues such as unclear video recordings or technical difficulties. Among the successful events, 99% (16,975/17,151) were validated by investigators as verified on-time dosing, indicating that the actual dosing time was within the allowed SAI time window and the dosing behavior recorded in the video was appropriate.

A total of 127 SAI days were analyzed, with over 90% (367/407) of participants consistently reporting dosing videos on all SAI days. The majority of SAI days (95%, 121/127) had more than 90% objective dosing data, with some days requiring additional verification via phone to ensure accurate data capture. This high rate of validated dosing (verified on-time dosing and verified deviated dosing) indicates that the system effectively supported the accurate SAI (Table 3 and Multimedia Appendix 1).

**Table 3:** Self-administration of the investigational medicinal product success rate by Cohort and by study day. Success rate (%)=(validated dosing events/planned SAIs^a^ in each study day) × 100.

Cohort	Study day																Cohort overall
	1	2	3	4	5	6	7	8	9	10	11	12	13	14	≥15		
1	N/A^b^	99	99	99	N/A	100	99	97	N/A	99	100	98	N/A	99	99	99	
2	93	100	N/A	N/A	N/A	92	100	N/A	100	100	100	N/A	N/A	N/A	N/A	98	
3	100	100	N/A	96	100	100	N/A	N/A	N/A	100	95	N/A	N/A	N/A	N/A	98	
4	100	100	N/A	N/A	N/A	N/A	N/A	N/A	N/A	N/A	N/A	100	100	100	100	100	
5	95	98	100	100	N/A	N/A	N/A	N/A	N/A	N/A	N/A	N/A	97	100	100	99	
6	83^c^	91	95	93	97	91	89^c^	90	94	92	93	95	91	93	95	93	
7	N/A	N/A	100	N/A	100	N/A	100	N/A	100	100	N/A	100	100	100	100	100	
8	0^c^	92	92	94	97	96	94	97	98	97	94	97	99	97	97	93	
9	N/A	N/A	87^c^	91	98	96	93	N/A	N/A	N/A	N/A	N/A	N/A	N/A	N/A	93	
10	N/A	N/A	N/A	100	98	100	100	100	N/A	N/A	100	N/A	N/A	N/A	N/A	100	
11	N/A	N/A	N/A	N/A	100	96	98	89^c^	96	N/A	N/A	79^c^	N/A	N/A	N/A	93	
12	N/A	100	100	100	N/A	N/A	N/A	100	100	100	100	96	100	98	100	99	
13	100	100	94	100	100	100	100	100	100	98	100	100	100	98	99	99	
14	N/A	100	100	N/A	100	100	N/A	100	100	N/A	100	N/A	N/A	N/A	N/A	100	
By study day	66	96	96	96	97	97	96	95	98	97	98	96	97	97	98	N/A	

^a^SAI: self-administration of the investigational medicinal product.

^b^N/A (not applicable) indicates SAI was not performed on the study day.

^c^Success rate is less than 90%.

### Trends in Dosing Time Deviations by Cohort

The pattern of PADEV by cohort appeared to be random and did not show any consistent trend depending on the characteristics of the IMP or the scheme of the SAI. For almost all SAI events, the SD of PADEV was greater than the mean, showing that dosing deviations per participant were highly variable around the planned dosing time. Cohort 10 had the earliest mean PADEV during the initial period at –29.6 (SD 65.8) minutes, and this trend remained consistent in subsequent periods, with a mean deviation of –27.2 (SD 65.9) minutes. Similarly, cohort 8 had the latest mean PADEV of 24.0 (SD 50.5) minutes during the initial period and maintained a similar pattern thereafter with a deviation of 27.6 (SD 53.1) minutes.

**Figure 2:**
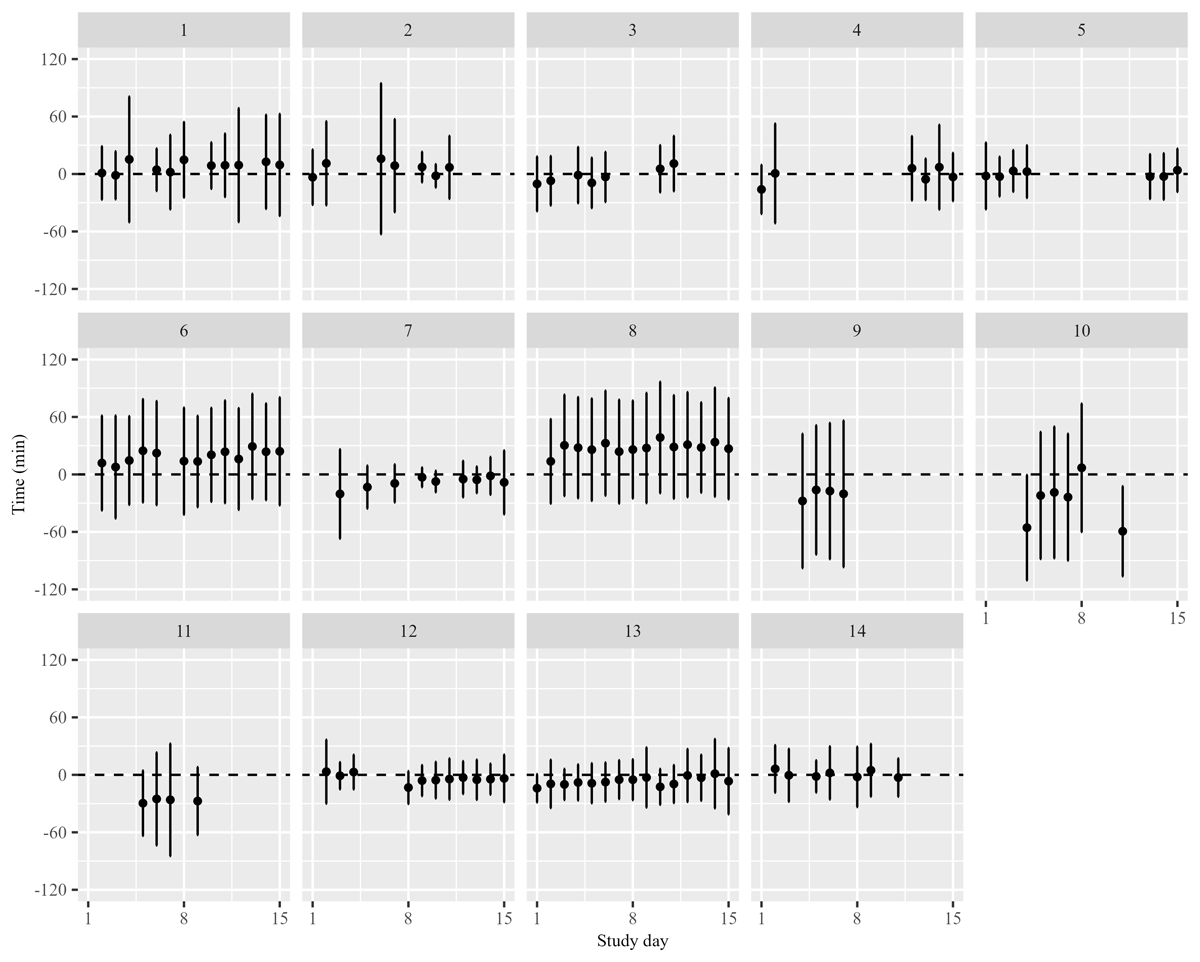
Distribution of the dosing time deviation by study day in each cohort. The distribution illustrates the trends in self-administration times among participants over the course of the study, demonstrating the extent to which dosing occurs earlier or later in comparison to on-time dosing (0 minutes).

A trend toward delayed dosing was observed in cohorts 1, 2, 6, 8, 12, and 14, especially during the initial period. For example, cohort 1 had a mean deviation of 5.0 (SD 44) minutes in the initial period, with subsequent deviations ranging from 1.9 (SD 38.9) minutes to 14.8 (SD 39.5) minutes. Cohort 6 had an initial mean deviation of 11.3 (SD 49.9) minutes, which increased to 21.5 (SD 54.5) minutes in the second half. Conversely, cohorts 3, 4, 5, 7, 9, 10, 11, and 13 tended to dose earlier than the planned time, and this trend was maintained over time. For example, cohort 3 had an initial mean deviation of –6.3 (SD 27.8) minutes, which was followed by a deviation of –2.7 (SD 27.5) minutes. Cohort 7 had an initial deviation of –14.3 (SD 30.9) minutes and remained at the same deviation of –8.3 (SD 32.4) minutes afterward (Figure 2 and Multimedia Appendix 2). While some SAIs were conducted at times that may be outside of the participants’ daily routine (after 10 PM and before 7 AM), the overall SAI pattern was not significantly different from the typical time of day.

Tests of comparison between reference (the first 3 study days in which SAIs were performed) and subsequent study days showed no significant differences in dosing time deviations for most cohorts. However, significant differences (*P*<.05) were observed on 13 of 79 (16%) days, indicating occasional deviations from the initial trend (Table 4). For example, cohort 12 had a significant difference on day 8 with a mean deviation of –13.3 (SD 17.1) minutes compared with 1.8 (SD 23.0) minutes in the initial period. The detailed results of the analysis are presented in Multimedia Appendix 2.

**Table 4:** Proportion of study days with the significant difference in mean dosing time from reference day.

Cohort	Proportion, % (n/N)^a^
1	0 (0/8)
2	0 (0/4)
3	25 (1/4)
4	0 (0/3)
5	0 (0/4)
6	40 (4/10)
7	0 (0/6)
8	9 (1/11)
9	0 (0/1)
10	67 (2/3)
11	0 (0/1)
12	13 (1/8)
13	25 (3/12)
14	0 (0/4)
Total	16 (13/79)

^a^Proportion (number of study days with a significant mean PADEV [difference between planned and actual dosing time] difference from the reference period/total study days with SAI excluding the reference period).

**Figure 3:**
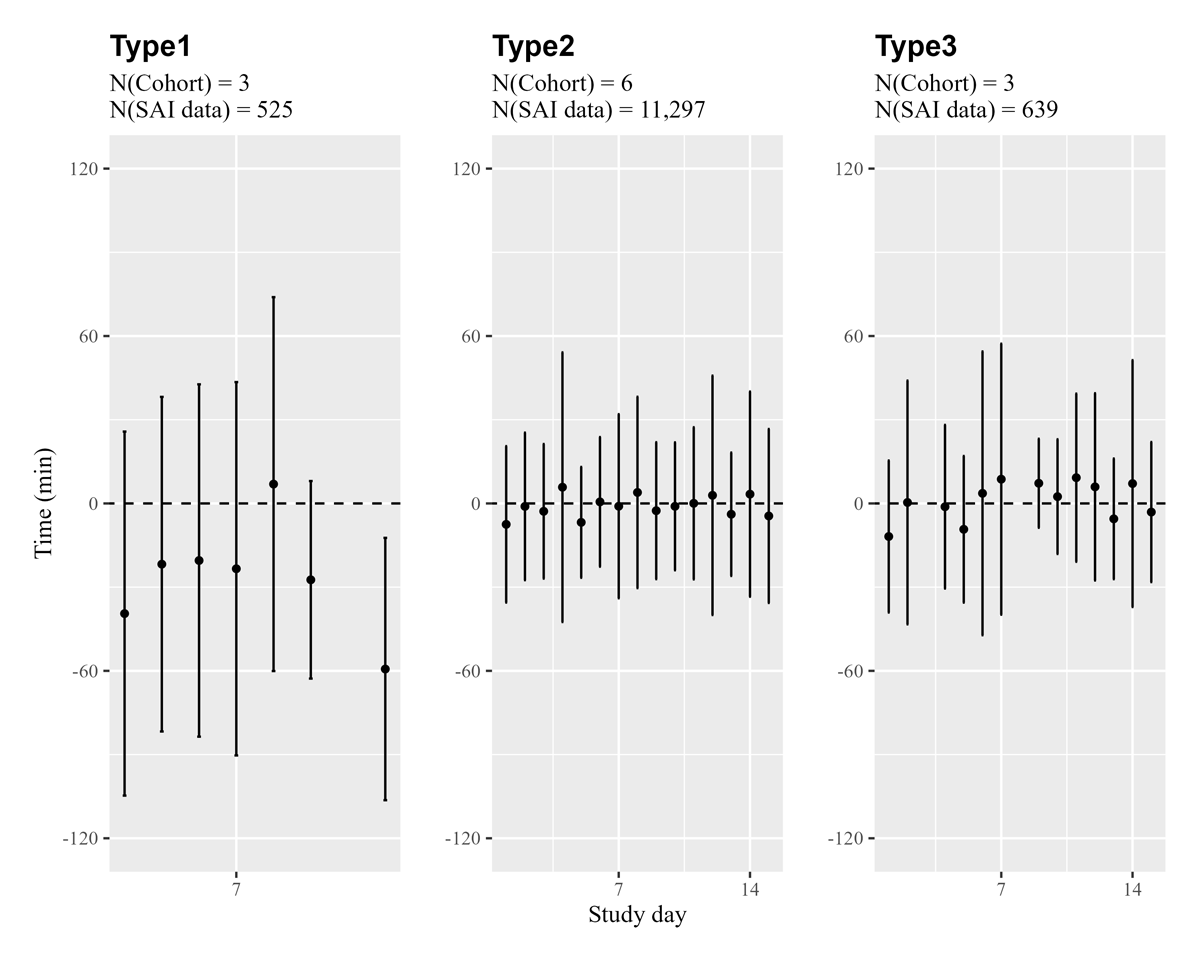
Trends in the dosing time deviation by push notification type. SAI: self-administration of the investigational medicinal product. Push notification types are as follows: type 1 (dosing notification only), type 2 (both dosing notification and dosing reminder), and type 3 (no push notifications). The distribution illustrates the trends in self-administration times for each type based on the push notifications sent through the VSMS. VSMS: video-based self-administration of the investigational medicinal product monitoring system.

For type 1 cohorts, which included Cohorts 9, 10, and 11 with a total of 525 SAI data points, the average PADEV was –24.9 (SD 63) minutes. This indicates a tendency to administer doses earlier than planned when only notifications were used. type 2 cohorts included cohorts 1, 5, 7, 12, 13, and 14; cohorts 6 and 8 were excluded due to potential bias in PADEV due to formulation differences (powder). The mean and SD of the PADEV was –3.5 (SD 31.3) minutes, and it should be noted that this type contained significantly more SAI events than the other types. This suggests that the SD of other types is likely overestimated compared to this type. In a mean difference test performed to account for this possibility (without the equal variance assumption), this type was significantly different from type 1, but not from type 3. The cohorts with no push notifications (type 3), including cohorts 2, 3, and 4 with 639 SAI data points, showed an average PADEV of –1.3 (SD 32.2) minutes. Despite the lack of notifications, these cohorts also maintained relatively small mean deviation, similar to type 2 (Table 5 and Figure 3).

**Table 5:** Distribution of dosing time deviation by push notification type and study day.

Study day	Deviation (minutes), mean (SD)		
	Type 1^a^ (n^d^=525)	Type 2^b^ (n^d^=11,297)	Type 3^c^ (n^d^=639)
1	N/A^e^	–7.5 (28)	–11.9 (27.2)
2	N/A	–1.1 (26.4)	0.4 (43.7)
3	N/A	–2.8 (24.1)	N/A
4	39.5 (65.2)	5.8 (48.3)	–1.2 (29.3)
5	–21.8 (60)	–6.8 (19.9)	–9.3 (26.3)
6	–20.5 (63.1)	0.6 (23.2)	3.6 (50.9)
7	–23.4 (66.9)	–1.0 (33.0)	8.7 (48.6)
8	6.9 (67)	3.9 (34.3)	N/A
9	–27.4 (35.4)	–2.6 (24.5)	7.2 (16)
10	N/A	–1.0 (22.9)	2.4 (20.6)
11	–59.4 (47)	0.0 (27.3)	9.2 (30.1)
12	N/A	2.9 (42.9)	5.9 (33.6)
13	N/A	–3.9 (22.1)	–5.5 (21.6)
14	N/A	3.3 (36.8)	7.1 (44.2)
15	N/A	–4.5 (31.2)	–3.1 (25.1)
Total period	–24.9 (63)	–3.5 (31.3)^f^	–1.3 (32.2)^g^

^a^Type 1 includes cohorts that received only dosing notification.

^b^Type 2 includes cohorts that received both dosing notification and dosing reminder.

^c^Type 3 includes cohorts that did not receive any push notifications.

^d^Number of self-administration events.

^e^N/A: not applicable.

^f^*P*<.001 on comparing with type 1.

^g^*P*<.001 on comparing with type 1 and *P*=.09 on comparing with type 2.

## Discussion

### Principal Findings

The cohort characteristics of this study highlight key considerations for the implementation and scalability of VSMS. In all cohorts, high adherence was mandatory to achieve the study objectives, and participants provided informed consent before participation. This is likely the primary reason for the relatively high adherence observed across cohorts compared with that observed in later clinical trials or real-world settings [5,26]. Nevertheless, the clear benefits of using a VSMS were consistent with previous studies of similar systems [16-18,20-25,27-30]. By providing visual evidence of SAI, the VSMS enabled SAI instead of direct observation, and the video-based, real-time management allowed investigators to monitor adherence during the dosing period and intervene in a timely manner to minimize noncompliance [17,23-25,31]. In this study, additional measures to improve the reliability of the data were proposed by clarifying the outcome categorization system of verified on-time dosing, verified deviated dosing, unverified dosing, and missed dosing. While VSMS increases the participant’s performance burden for the SAI itself, it reduces the inconvenience of site visits for dosing and minimizes the potential for participant dropout due to nonadherence [6,27,32], expanding the feasibility of a variety of clinical studies that require full adherence and its evidence. Conversely, the use of VSMS in studies that only investigate the percentage of planned doses actually taken and do not require thorough evidence of adherence will need to be determined on a cost-benefit basis.

Adherence in cohorts with SAI using VSMS was similar to that observed in repeated-dose early-phase clinical trials based on direct observation (where almost 100% adherence is required for participants). Furthermore, the concept of adherence extends not only to the performance of SAIs, but also to the use of the provided system to record SAI behavior and transmit it to the researcher. Therefore, high adherence in studies using VSMS indicates that the essential requirements of the system have been met, which (1) technical reliability: the system operated without significant technical errors, ensuring that data capture and processing was accurate and reliable; (2) participant acceptability: participants were able to use the mobile-based application without difficulty, which is critical to maintaining high levels of compliance in clinical trials; and (3) comprehensiveness for investigators: the system provided sufficient information in an appropriate manner to enable investigators to make informed decisions regarding the validation of SAI. Along with the intuitive user interface, user acceptability can be attributed in part to the initial training during the first visit, including a mock SAI session.

The study observed that SAI behaviors established early in the trial were generally maintained throughout the study period. Only 16% (13/79) of events showed statistically significant differences in dosing times compared with the initial SAI events. When using dosing notifications alone, a deviation toward faster SAI times was observed compared with using dosing reminders together or no push notifications at all. The use of dosing notification alone was associated with a faster deviation to SAI time compared with the use of dosing reminder or no push notification at all. Even accounting for the difference in the number of events in each group, the variability in deviation was greatest for dosing notification alone. The fact that the distribution of deviations was similar between no push notification group and both dosing notification and reminder group suggests that participants were highly motivated to adhere to their planned dosing times. In this situation, using only the dosing notification would have resulted in participants choosing to perform SAI before they forgot to do it, and there would have been significant interparticipant variation in this choice. Although push notifications were preset and remained constant throughout the study period in the cohorts included in these studies, given the nature of SAI behavior and the impact of push notifications, it is possible that better compliance could be achieved by adjusting push notifications based on the initial SAI pattern of the cohort. Particularly, it is considered necessary to modify the strategy of push notifications by identifying the tendencies of the participants at the beginning of the SAI period when only dosing notification is planned.

The VSMS used in this study is compatible with any type of mobile device with video recording and wireless transmission capabilities. As an alternative to installing the VSMS application on a participant’s smartphone, a separate mobile device capable of performing VSMS could be provided for the entire study period to conduct the SAI. Some participants in the cohort included in this study participated in this manner. This fact means that the participant or patient does not necessarily need to own a smartphone and agree to install the VSMS application in order to use VSMS. In other words, the usefulness of VSMS depends on the participant’s willingness to perform the SAI and the appropriate use of the VSMS application, rather than the ownership of a mobile device.

### Limitations

The findings were derived from a population with limited demographic characteristics. The usefulness of VSMS is likely to be contingent upon the participant’s or patient’s capability to use mobile apps and the mobile environment in which they reside. It is expected that prospective evaluations in larger and more diverse populations, including different participant characteristics in terms of age, dosing regimens, or indications, will provide even more reliable evidence.

### Conclusions

VSMS proved to be an effective tool for obtaining dosing information with accuracy comparable to direct observation, even in remote situations. The potential to minimize adherence deterioration through various alarm features and appropriate investigator or observer intervention was also identified. In many research or clinical situations where participant adherence is essential, but direct observation is difficult, VSMS may be a key element in obtaining dosing information. Proper usage of participants’ smartphones or other mobile devices and previous training of participants and observers may improve the accuracy of SAI recording via VSMS. It is expected that the usage and usefulness of VSMS will be continuously improved through the accumulation of experience in various medical fields.

## Appendix

Multimedia Appendix 1 The proportion of SAI (self-administration of the investigational medicinal product) outcome by category on each cohort.

Multimedia Appendix 2 The results of the comparison between the mean PADEV (difference between planned and actual dosing time) on the reference study day and the mean PADEV on each subsequent study day, along with the distribution of daily PADEV.

## Abbreviations

IMP: investigational medicinal product
IRB: institutional review board
PADEV: difference between planned and actual dosing time
SAI: self-administration of the investigational medicinal product
VSMS: video-based self-administration of the investigational medicinal product monitoring system
