# Correction: Participant Adherence in Repeated-Dose Clinical Studies Using Video-Based Observation: Retrospective Data Analysis

**DOI:** 10.2196/79507

**Published:** 2025-07-30

**Authors:** Jihong Song, Sungpil Han, Suein Choi, Jonghyuk Lim, Byeong Yeob Oh, Dongoh Shin, Seunghoon Han

**Affiliations:** 1 Department of Pharmacology College of Medicine The Catholic University of Korea Seoul Republic of Korea; 2 Department of Clinical Pharmacology and Therapeutics Seoul St. Mary’s Hospital, College of Medicine The Catholic University of Korea Seoul Republic of Korea; 3 CareSquare, Inc. Seoul Republic of Korea

In “Participant Adherence in Repeated-Dose Clinical Studies Using Video-Based Observation: Retrospective Data Analysis” (JMIR JMIR Mhealth Uhealth 2025;13:e65668), the authors noted three errors.

- The order of authors was incorrectly listed in the originally published article. This has been changed from:

Seunghoon Han, MD, PhD; Jihong Song, BSN; Sungpil Han, MD, PhD; Suein Choi, MD, PhD; Jonghyuk Lim, BE; Byeong Yeob Oh, BBA; Dongoh Shin, MEng

To:

Jihong Song, BSN; Sungpil Han, MD, PhD; Suein Choi, MD, PhD; Jonghyuk Lim, BE; Byeong Yeob Oh, BBA; Dongoh Shin, MEng; Seunghoon Han, MD, PhD

- In the subsection of “Methods” titled “The System Overview,” casing type was changed from:

(2) Verified Deviated Dosing, (3) Unverified Dosing, (4) Missed Dosing

To:

(2) Verified deviated dosing, (3) Unverified dosing, (4) Missed dosing

- The values in the final column of Table 2, “Allowed time window for SAI (min),” were adjusted to more accurately represent the intended time windows. These values have now been provided in a range rather than a single negative value, and the table will appear as follows:

**Table 2:** Self-administration of the investigational medicinal product-specific characteristics by cohort.

Cohort	SAI^a^ schedule				Dosing notification (min before the planned dosing time)	Dosing reminder (min after the planned dosing time)	Allowed time window for SAI (min)
	SAI day	Number of total SAI days	Number of SAIs per day	Planned dosing time^b^			
1	2-4, 6-8, 10-12, 14-16, 18, 19	14	1	9 AM	–60, –30, 0	15, 30, and 45	–60 to 60
2	1, 2, 6, 7, 9-11	7	1	9 AM	N/A^c^	N/A	–60 to 60
3	1, 2, 4-6, 10, 11	7	1	9 AM	N/A	N/A	–60 to 60
4	1, 2, 12-15, 17-20, 24, 25	12	1	9 AM	N/A	N/A	–60 to 60
5	1-4, 13-17, 21-23	12	1	9 AM	–60, –30, 0	15, 30, and 45	–60 to 60
6	1-32	32	2	Variable^d^	–60, –15, 0	60 and 110	–120 to 120
7	3, 5, 7, 9, 10, 12-14, 16-21, 23-30, 32-45, 47-60, 62-75, 77-90	78	1	9 AM	–30, –5, 0	10, 20, 165, and 665	–180 to 180
8	1-28	28	2	Variable^d^	–60, –10, 0	60, 180, and 350	–120 to 360
9	3-7	5	2^e^, 3	8 AM, 2 PM, and 8 PM	–30, 0	N/A	–120 to 120
10	4-8, 11	6	2^e^, 3	8 AM, 2 PM, and 8 PM	–30, 0	N/A	–120 to 120
11	5-9, 12	6	2^e^, 3	8 AM, 2 PM, and 8 PM	–30, 0	N/A	–120 to 120
12	2-4, 8-96	92	1^e^, 2^e^, 4	1 AM, 9 AM, 9:10 AM, 5 PM	–10, 0	10	N/A
13	1-89, 92-96	94	2^e^, 3	1 AM, 9 AM, 5 PM	–10, 0	10	N/A
14	2, 3, 5, 6, 8, 9, 11	7	1	9 AM	–30, 0	30	–60 to 60

^a^SAI: self-administration of the investigational medicinal product.

^b^The presented data refer to the reference point for the planned dosing time (eg, 9 AM) in each cohort, with minor variability across participants (eg, 9:02 AM, 9:04 AM, 9:06 AM, etc).

^c^N/A: not applicable.

^d^The planned dosing times vary across participants, and no common reference point can be provided.

^e^SAI event and dosing by the investigator on the same day.

The correction will appear in the online version of the paper on the JMIR Publications website, together with the publication of this correction notice. Because this was made after submission to PubMed, PubMed Central, and other full-text repositories, the corrected article has also been resubmitted to those repositories.

